# Functional outcome and muscle wasting in adults with tetanus

**DOI:** 10.1093/trstmh/trz055

**Published:** 2019-07-24

**Authors:** Truong N Trung, Nguyen V T Duoc, Le T H Nhat, Lam M Yen, Nguyen V Hao, Nguyen T Truong, Ha T H Duong, Duong B Thuy, Nguyen T Phong, Le V Tan, Zudin A Puthucheary, C Louise Thwaites

**Affiliations:** 1 Hospital for Tropical Diseases, 764 Vo Van Kiet, Quan 5, Ho Chi Minh City, Viet Nam; 2 Oxford University Clinical Research Unit, Hospital for Tropical Diseases, 764 Vo Van Kiet, Quan 5, Ho Chi Minh City, Viet Nam; 3 University of Medicine and Pharmacy, Hong Bang, Phuong 11, Quan 5, Ho Chi Minh City, Viet Nam; 4 William Harvey Research Institute, Barts and London School of Medicine and Dentistry, Queen Mary University of London, London, UK; 5 Adult Critical Care Unit, Royal London Hospital, London, UK; 6 Centre for Tropical Medicine and Global Health, University of Oxford, Nuffield Department of Medicine Research Building, University of Oxford, Old Road Campus, Roosevelt Drive, Headington, Oxford OX3 7FZ, UK

**Keywords:** functional outcome, hospital-acquired infection, muscle mass

## Abstract

**Background:**

In many countries, in-hospital survival from tetanus is increasing, but long-term outcome is unknown. In high-income settings, critical illness is associated with muscle wasting and poor functional outcome, but there are few data from resource-limited settings. In this study we aimed to assess muscle wasting and long-term functional outcome in adults with tetanus.

**Methods:**

In a prospective observational study involving 80 adults with tetanus, sequential rectus femoris ultrasound measurements were made at admission, 7 days, 14 days and hospital discharge. Functional outcome was assessed at hospital discharge using the Timed Up and Go test, Clinical Frailty Score, Barthel Index and RAND 36-item Short Form Health Survey (SF-36) and 3 and 6 months after discharge using the SF-36 and Barthel Index.

**Results:**

Significant muscle wasting occurred between hospital admission and discharge (p<0.01), particularly in severe disease, where a median 23.49% (interquartile range 10.01–26.07) reduction in rectus femoris cross-sectional area occurred in those with severe (Ablett grades 3 and 4) disease. Muscle mass at discharge was related to objective and subjective measures of physical and emotional function at discharge and 3 and 6 months after discharge. In patients >70 y of age, functional recovery at 6 months was reduced compared with younger patients. Hospital-acquired infection and age were risk factors for muscle wasting.

**Conclusions:**

Significant muscle wasting during hospitalization occurred in patients with tetanus, the extent of which correlates with functional outcome.

Tetanus remains a significant problem in many low- and middle-income countries (LMICs), responsible for up to 50 000 deaths annually.^1^ However, even in resource-limited settings, with good care, survivorship from tetanus is often >90%.^2–4^ The functional outcome of these survivors is unknown and while there are limited data regarding neurodevelopmental outcome following neonatal tetanus, there are no studies of long-term outcome in adults.^5^ In many countries, as a result of successful childhood and maternal vaccination programs, tetanus predominantly affects working-age men,^1^ thus the consequence of limited functional recovery may have far-reaching social and economic impact.

In high-income countries, intensive care unit (ICU) survivorship is frequently associated with significant disability.^6^ ICU-acquired weakness and muscle wasting are potentially modifiable contributors to this, both in the short term (length of stay, mortality, length of mechanical ventilation) and long term (mortality, continued functional decline, discharge to a rehabilitation centre).^7–10^ Bed rest, duration of mechanical ventilation, inflammatory response, corticosteroids and glycaemia have all been implicated in muscle wasting and weakness, although the role of other factors, such as neuromuscular blocking agents, remains unclear.^11–13^ The introduction of point-of-care ultrasonography means that quantification of muscle wasting in low-resource settings is now feasible. Ultrasound measurement of quadriceps femoris has been shown to be a reliable method for measuring muscle mass. It can be performed in sedated or unconscious patients and has good inter- and intra-observer reliability.^10^ The cross-sectional area of the quadriceps has been shown to correlate with functional outcome and a linear relationship between ultrasound measurement of rectus femoris cross-sectional area (RF-CSA) and quadriceps strength has been demonstrated.^9,10^

Patients with tetanus may be particularly at risk of muscle wasting and poor outcome due to long periods in the ICU and prolonged use of mechanical ventilation and muscle relaxants.^3^ In this study we aimed to characterize functional outcome and changes in muscle quantity in a large cohort of patients with tetanus. Much previous work on muscle wasting has been confounded by population heterogeneity. By recruiting only those with tetanus in this study, we were able to examine muscle wasting in a single, clearly defined disease and a population with low comorbidity or other factors likely to introduce bias.

## Methods

This was a prospective observational study, approved by the Scientific and Ethics Committee of the Hospital for Tropical Diseases and the Oxford Tropical Research Ethics Committee (OxTREC). All subjects, or their representatives, gave written informed consent prior to enrolment according to Vietnamese Ministry of Health policy and good clinical practice guidelines. Patients were enrolled in the adult ICU at the Hospital for Tropical Diseases, Ho Chi Minh City between August 2016 and March 2017. The hospital is an 850-bed tertiary referral centre for infectious diseases, serving southern Vietnam (population 44 million). Its adult ICU is a 20-bed medical ICU with facilities for mechanical ventilation and haemofiltration. Tetanus management in our ICU follows a standard management protocol. Muscle spasms are controlled with benzodiazepines, with magnesium sulphate and neuromuscular blocking agents as second-line agents following tracheostomy. Autonomic nervous system dysfunction is treated with magnesium sulphate. Steroids are not used in treatment. All patients received intramuscular equine antitoxin and metronidazole.

Adult patients ≥16 y of age with a clinical diagnosis of generalized tetanus and within 48 h of ICU admission were eligible for study entry. Tetanus was diagnosed according to clinical criteria detailed in the Hospital for Tropical Diseases Guidelines Volume 6, including generalized muscle rigidity and/or muscle spasm, without alteration in consciousness or fever at the onset of symptoms.^14^ Those failing to give informed consent, unable to walk unaided prior to hospitalization or with contraindication to ultrasound were excluded. A sample size of 80 patients was estimated to detect a 15% reduction of RF-CSA between hospital admission and discharge with 95% confidence and 5% error. For logistical reasons, patients admitted on weekends or during shifts when study staff were not available were not enrolled.

Baseline clinical and demographic data were recorded and daily clinical assessments of patients were performed throughout the ICU stay. The duration of mechanical ventilation, dose and duration of muscle relaxant drugs and hospital-acquired infection events were recorded. Both midazolam and diazepam were used and doses recorded. Generally, practice in our unit is to give midazolam to more severe patients; however, issues such as cost and availability may also influence benzodiazepine prescription. Tetanus severity stage was classified using the modified Ablett grades,^15,16^ where mild tetanus was defined as Ablett grades 1 and 2 and severe tetanus as grades 3 and 4. The Ablett grade may change during hospitalization and is intrinsically linked to events such as mechanical ventilation, sedation and hospital-acquired infection, therefore the baseline Tetanus Severity Score was used in risk factor analyses.^15,16^

Muscle mass was determined using B-mode ultrasound measurement of RF-CSA using an M-Turbo ultrasound machine with a standard 5–2 MHz C60Xi transducer (FUJIFILM SonoSite, Bothell, WA, USA). Measurements were performed according to a standard operating procedure previously described.^10^ Briefly, patients were laid in a supine position with legs in a neutral rotation and passive extension. Measurements were taken from the right leg at the midpoint between the anterior superior iliac spine and patella upper pole. This position was marked for subsequent assessments. The transducer was placed perpendicular to the skin and in a transverse position (perpendicular to the thigh’s long axis). An excess of ultrasound gel was placed on the skin to prevent skin depression by the transducer. Ultrasound measurements were taken at baseline, 7 and 14 d and at hospital discharge. For each time point, measurements were taken in triplicate and the average was calculated and used for analyses. Measurements were carried out by three operators. Operators were deemed competent to perform study measurements if intra- and interobserver variation of <5% was attained from a test series of 30 images.

At hospital discharge, functional outcome was assessed using objective and subjective measures: RAND 36-item Short Form Health Survey (SF-36), Barthel Index, Timed Up and Go test and Clinical Frailty Score.^17–20^ The SF-36 has previously been translated and used in Vietnamese subjects, but there are no normative data in this population.^21^ At 3 and 6 months following hospital discharge, Barthel Index and SF-36 questionnaires were performed by telephone.

All data were recorded in standard case record forms and transferred to a secure database. Statistical analysis was performed with R software (Version 3.5.1, R Foundation for Statistical Computing, Vienna, Austria). Data are presented as median (interquartile range [IQR]). Baseline and clinical characteristics were compared using Mann–Whitney U and χ^2^ tests. Median muscle mass and age-related functional outcome scores were compared using the Mann–Whitney U test and multiple comparisons of these adjusted using the Bonferroni correction. All longitudinal measurements of muscle mass at baseline, 7 and 14 d and hospital discharge were included in the analysis. The association between functional outcome and muscle mass was analysed using a linear regression model. For reliability of the SF-36, Cronbach’s α was calculated for each domain score using ‘psy’ in the R package.

To investigate the effect of hospital-acquired infection, ventilation, neuromuscular blocking agents and magnesium sulphate on muscle wasting, a linear mixed effects model was used. This model can incorporate multiple repeated measurements at varying time points, eliminate effects of between-subject variability and incorporate non-independent and time-dependent covariates. RF-CSA was the outcome variable, modelled with time from ICU admission and hospital-acquired infection as time-dependent covariates and mechanical ventilation, neuromuscular blocking agents and magnesium sulphate as time-independent covariates. The relationship between time since admission to the ICU and RF-CSA was modelled a flexible way using restricted cubic splines with three knots [7,14,21]. A random patient-specific intercept and slope was included to account for individual heterogeneity. The model was adjusted for potential confounding covariates of age, comorbidity and tetanus severity using the Tetanus Severity Score, a baseline score constructed from clinical features on admission.^16,22^

## Results

A total of 80 patients were recruited to the study between August 2016 and March 2017 (Supplementary Figure 1). All patients had ultrasound assessments 7 days after enrolment but two patients with mild disease were discharged from the hospital before day 14 scans (these patients had a discharge scan but no day 14 scan). One patient was transferred to another hospital after 15 d and subsequently died in that hospital, leaving 79 patients with complete assessments at hospital discharge. One patient, reported to have recovered completely at 3 months, died suddenly before the 6-month follow-up, following a short febrile illness. Seven patients were lost to follow-up with 75 patients completing functional outcome assessments at 3 months and 71 at 6 months.

### Muscle mass

**Figure 1.**
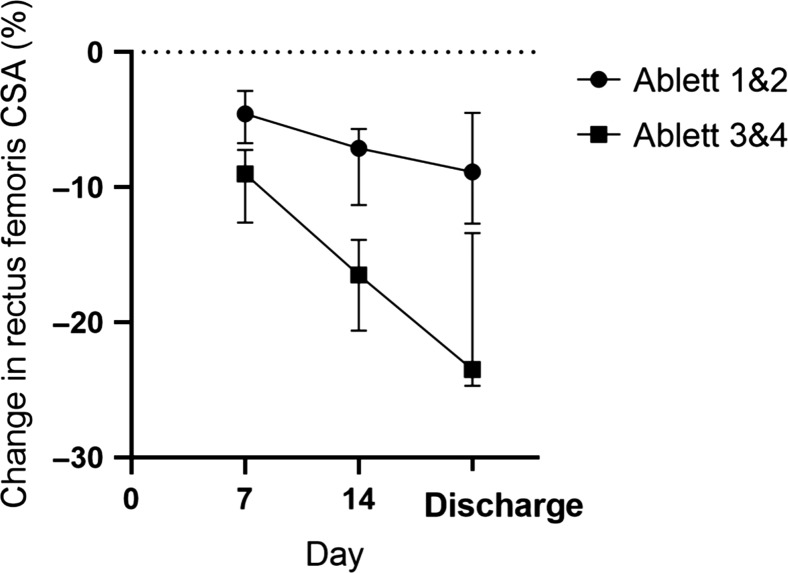
Median (95% confidence interval) change in RF-CSA (CSA) compared with baseline according to Ablett severity. Ablett grades 1 and 2: mild disease with spasms not interfering with respiration; Ablett grades 3 and 4: severe disease with spasms interfering with respiration with or without autonomic nervous system dysfunction.

Summary baseline data for subjects are given in Table 1. Overall, compared with baseline, RF-CSA was reduced by a median 6.63% (IQR 2.73–13.73) at day 7, 11.94 % (IQR 5.41–21.1) at day 14 and 12.3% (IQR 4.7–23.57) at discharge. The linear mixed effects model showed a significant reduction in muscle mass between admission and discharge (p<0.01; Table 2, Supplementary Figure 2). Muscle wasting was greatest in patients with severe disease (Ablett grades 3 and 4) (Figure 1). Seven days after hospitalization, patients with mild disease (Ablett grades 1 and 2) had lost a median 4.57% (IQR 1.81–10.20) RF-CSA compared with those with Ablett grades 3 and 4 (9.01% [IQR 3.95–16.85]) (n=80, p=0.15). At day 14, the median reduction was 7.12% (IQR 3.11 −17.5) in those with mild disease compared with 16.54% (IQR 13.23−24.10) for those with Ablett grades 3 and 4 (n=78, p<0.01), and at hospital discharge there was a median 8.86% (IQR 2.9–16.54) decrease in patients with mild disease compared with 23.49% (IQR 10.01–26.07) with Ablett grades 3 and 4 (n=80, p=0.03).

**Table 1.** Patient characteristics

Characteristics	All patients (n=80)	Ablett grades 1 and 2 (n=48)	Ablett grades 3 and 4 (n=32)	p-Value
Baseline				
Age (years), median (IQR)	49 (35–59.5)	46 (33.5–57)	51.5 (41.5–64.5)	0.15
Male:female ratio, n:n	64:16	9:39	7:25	0.73
Tetanus Severity Score, median (IQR)	−1 (−6–2)	−2 (−5–0)	1 (−2.25–5.25)	<0.01
SOFA score, median (IQR)	0 (0–0)	0 (0–0)	0 (0–0)	0.15
SOFA >0, n (%)	7 (9)	2 (4)	5 (16)	0.15
Subjects with comorbidity, n (%)	22 (27.5)	11 (22.9)	11 (34.4)	0.26
Subjects with hospital-acquired infection, n (%)	28 (35)	6 (12.5)	22 (69)	<0.01
Diabetes, n (%)	8/80 (10)	5 (3)	3 (9.7)	0.92
Hypertension, n (%)	18/80	10 (0.21)	8 (25)	0.66
Previous myocardial infarction, n (%)	1/80 (1.3)	0 (0)	1 (3.1)	0.83
Congestive heart failure, n (%)	0 (0)	0 (0)	0 (0)	1
Peripheral vascular disease, n (%)	1 (1.3)	0 (0)	1 (3.1)	0.83
Cerebrovascular disease, n (%)	1 (1.3)	1 (2.1)	0 (0)	1
Chronic obstructive pulmonary disease, n (%)	0 (0)	0 (0)	0 (0)	1
Chronic liver disease, n (%)	1 (1.3)	1 (2.1)	0 (0)	1
Charlson Comorbidity Index, median (IQR)	0 (0–1)	0 (0–0)	0 (0–1)	0.32
Clinical features				
Hospital length of stay (days), median (IQR)	25 (20–33)	21 (18–25)	34 (30–42.5)	<0.01
ICU length of stay (days), median (IQR)	14 (7–23)	8 (5–12)	25 (20–30)	<0.01
Body mass index, median (IQR)	20.95 (19.14–23.70)	21.16 (19.13–24.07)	20.81 (19.51–23.46)	0.71
Total diazepam (mg), median (IQR)	437.5 (211.3–955)	565 (293.8–858.8)	235 (126.25–2362.5)	<0.01
Total midazolam (mg), median (IQR)	220 (0–1771)	0 (0–539)	1602.5 (0–2603)	<0.01
Total pipecuronium (mg), median (IQR)	224.4 (0–542.4)	0 (0–0)	493 (312–604)	<0.01
Total magnesium (g), median (IQR)	0 (0–0)	0(0–0)	24 (0–252)	<0.01
Mechanical ventilation, n (%)	32 (40)	2 (42)	30 (94)	<0.01
Duration of mechanical ventilation (days), median (IQR)	0 (0–15)	0 (0–0)	16.5 (13–23)	<0.01
Renal replacement therapy, n (%)	0 (0)	0 (0)	0 (0)	1
In-hospital deaths, n (%)	0 (0)	0 (0)	0 (0)	1
180-d deaths, n (%)	2 (2.5)	0 (0)	2 (6.2)	0.08
Lost to follow-up at 3 months, n (%)	3 (3.8)	3 (6.25)	0 (0)	0.15
Lost to follow-up at 6 months, n (%)	7 (8.8)	5 (10.4)	2 (6.3)	0.52

**Table 2.** Risk factors for muscle wasting from linear mixed effects model. The model was adjusted by time in a non-linear pattern (boundary knots not shown)

Risk factors	Coefficient*	95% CI	p-Value
Tetanus Severity Score	0.01	−0.10 to −0.12	0.90
Comorbidity	0.49	−0.67 to −1.64	0.42
Diabetes	−0.19	−1.70 to −1.36	0.81
(Age 17)/5	−0.44	−0.60 to −0.28	<0.01
Hospital-acquired infection	−0.48	−0.81 to −0.15	<0.01
Mechanical ventilation	0.16	−0.45 to 0.79	0.61
Pipecuronium	−0.43	−1.14 to 0.26	0.23
Magnesium sulphate	0.04	−0.46 to 0.55	0.66

Tetanus Severity Score predicts risk of adverse outcome from −8 (low risk) to 46 (high risk). Comorbidity, diabetes, hospital-acquired infection and mechanical ventilation are binary variables. Pipecuronium and magnesium sulphate are total drugs given in milligrams and grams, respectively.

*Coefficient is the regression coefficient of the linear mixed effects model representing the change in RF-CSA (in cm^2^) following 1 unit increase in the variable score. For example, hospital-acquired infection is associated with a 0.48 cm^2^ decrease in RF-CSA. For age, starting at age 17 y, a 5-y increase in age is associated with a 0.44 cm^2^ decrease in RF-CSA.

CI: confidence interval.

Thirty-nine patients with mild tetanus and four patients with severe tetanus were discharged from the ICU within 3 d of a day 7 or day 14 rectus femoris ultrasound measurement. At ICU discharge, these patients had lost a median 9.07% (IQR 3.27–17.32) of RF-CSA compared with baseline. By hospital discharge, no recovery of RF-CSA occurred, with area a median 9.91% (IQR 3.93–15.01) less than baseline (p=0.77). The median time between ICU discharge and hospital discharge ultrasound measurements was 12 d (IQR 9.5–16).

### Risk factors for muscle wasting

Using the linear mixed effects model, accounting for baseline severity and time since ICU admission, hospital-acquired infection and age were identified as independent risk factors for muscle wasting (Table 2; p<0.01 for both) but not the use of non-depolarizing neuromuscular blocking agents. A total of 29 patients developed hospital-acquired infections during the study: 12 with ventilator-associated pneumonia, 13 with urinary tract infection, 12 with bloodstream infection and 13 with ‘other’ infections (8 patients were treated for two or more infections). The majority of infections were treated with carbapenem antibiotics (20 patients), vancomycin (8 patients), fluroquinolones (9 patients) or third-generation cephalosporins (3 patients).

### Functional outcomes

**Figure 2.**
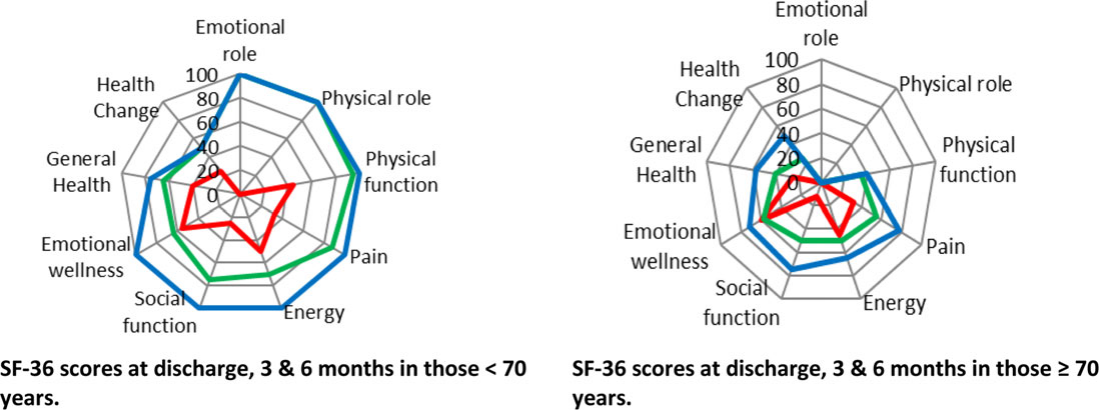
SF-36 scores at hospital discharge (red line) and 3 months (green) and 6 months (blue) after discharge. The figure shows different domains: physical functioning, bodily pain, role limitations due to physical health problems, role limitations due to personal or emotional problems, emotional well-being, social functioning, energy/fatigue, general health perceptions and single-item perceived change in health. The left panel shows scores in those <70 y of age and the right panel shows scores in those ≥70 y of age.

Functional outcome measures are shown in Table 3. The SF-36 Cronbach’s α showed good reliability, as shown in Supplementary Table 1. Both the Barthel Index and SF-36 domain scores improved by 3 and 6 months compared with discharge (Table 3). While overall scores improved by 3 and 6 months, in the subgroup of patients ≥70 y of age, functional recovery was significantly less than in younger individuals. At 3 and 6 months, all SF-36 domain scores were lower in patients ≥70 y of age than in younger patients (p≤0.05 for all; Figure 2). Similarly Barthel Index scores were lower in older patients (median 80 [IQR 74–85] vs 10 [IQR 100–100] at 3 months; median 80 [IQR 80–96] vs 100 [IQR 100–100] at 6 months; p<0.01 for both).

**Table 3.** Functional outcome scores at hospital discharge and 3 and 6 months

Measurement	Hospital discharge (n=79)	3 months (n=75)	6 months (n=71)
Clinical Frailty Score,^a^ median (IQR)	3 (2–4.5)	–	–
Timed Up and Go (s),^b^ median (IQR)	14 (11–20)	–	–
Barthel Index,^c^ median (IQR)	100 (80–100)	100 (100–100)	100 (100–100)
SF-36^d^			
Physical functioning, median (IQR)	40 (12.5–77.5)	95 (65–100)	100 (85–100)
Role limitation due to physical health, median (IQR)	0 (0–0)	100 (0–100)	100 (100–100)
Role limitation due to emotional problems, median (IQR)	0 (0–0)	100 (0–100)	100 (100–100)
Energy/fatigue, median (IQR)	50 (30–55)	60 (50–100)	95 (80–100)
Emotional well-being, median (IQR)	60 (51–64)	60 (56–96)	100 (80–100)
Social functioning, median (IQR)	25 (25–37.5)	75 (62.5–100)	100 (87.5–100)
Pain, median (IQR)	32.5 (22.5–43.75)	32.5 (22.5–43.75)	100 (92.5–100)
General health, median (IQR)	50 (30–55)	65 (50–72.5)	70 (65–95)
Health change, median (IQR)	25 (12.5–25)	25 (12.5–25)	50 (50–75)

^a^Clinical Frailty Score is a 7-point scale where 7 represents most frail [18].

^b^Timed Up and Go measures the time in seconds for an individual to stand up from a seated position, walk 3 m, turn around, return and sit down [16].

^c^Bathel Index indicates the ability to function independently in activities of daily living with a total score of 100, where 100 represents fully independent [17].

^d^SF-36 individual domain scores presented on a scale of 0–100, where 100 represents high function [19].

The relationship between functional outcomes and muscle wasting is shown in Supplementary Tables 2 and 3. Lower RF-CSA at hospital discharge was associated with worse functional outcome scores at hospital discharge as well as 3 and 6 months after discharge. Specifically, at hospital discharge, lower RF-CSA was associated with a statistically significant slower Timed Up and Go, increased Clinical Frailty score, lower Barthel Index and lower SF-36 physical functioning domain scores. A statistically significant association remained between RF-CSA measurements at hospital discharge and Barthel Index scores 6 months after discharge. RF-CSA measurements at hospital discharge were also statistically significantly associated with lower SF-36 scores in all domains except emotional wellness 6 months after discharge.

## Discussion

We investigated muscle mass loss and subsequent functional impairment in patients suffering from tetanus in a resource-limited setting. Mortality in our cohort was low, consistent with previously reported mortality at our centre. We attribute this largely to the expertise of a specialized centre, young age and limited comorbidity of the subjects. Nevertheless, in this study we found notable functional impairment at hospital discharge in survivors, with significant muscle loss during hospitalization, the extent of which was related to disease severity, age and the acquisition of hospital-acquired infection. Muscle mass at discharge was related to both objective and subjective measures of physical function.

Patient heterogeneity has been identified as one of the difficulties in understanding muscle wasting.^23^ Data from our study are derived from patients with a homogeneous non-inflammatory pathology, limited comorbidities, a common treatment protocol and within a narrow age range. Although we did not include a control group of other critically ill patients from our setting, our cohort demonstrated similar magnitudes and rates of reduction in RF-CSA to those described in mixed Western ICU populations and greater than those observed in healthy immobilized subjects.^10,24,25^ Our additional data with 14- and 21-d measurements demonstrates ongoing muscle mass loss into the second and third week of ICU stay.

Despite the nature of our study setting, we were able to contact the majority of patients after discharge, gaining insight into long-term outcomes following tetanus and the role of muscle mass. Low muscle mass at discharge was associated with poorer physical outcomes using objective (Timed Up and Go test, Barthel Index) and subjective (SF-36) measures. Further, low muscle mass at discharge was associated with increased frailty. While increased frailty has been noted in young critical care survivors, identification of the relationship with muscle mass has not occurred previously.^26^

Encouragingly in our cohort, most patients reported good recovery by 6 months, much of which occurred by 3 months. In healthy populations, spontaneous recovery of muscle cross-sectional area has been shown to occur rapidly following cessation of immobilization, but we found no evidence of recovery of RF-CSA between ICU and hospital discharge.^27^ While the reasons for our finding are unclear, suboptimal nutrition and lack of rehabilitation may be important factors, warranting further investigation.

Despite our cohort being younger than others studied, age was noted as a risk factor for increased muscle wasting.^10,22^ Comorbidities were not noted to be a risk factor, and these were of low frequency and mild relative to Western populations. In other ICU populations, rates of muscle wasting and weakness are associated with the degree of multi-organ failure.^22,28^ This was not seen in our population, likely as a result of the lack of organ failure. There was a relationship between disease severity, indicated by the Ablett grade, and increased wasting. However, Ablett grade changes with time and is defined by respiratory and autonomic complications, and thus is also linked to possible risk factors. In risk factor analysis, using a validated baseline disease severity score (Tetanus Severity Score), no relationship between severity and wasting was seen.^16^ It is therefore likely that the increased muscle wasting shown by the Ablett grade was related to differences in treatment or complications in the more severe patients.

Our finding that hospital-acquired infection is a risk factor for muscle wasting is novel, but is consistent with present understanding regarding the aetiology of muscle wasting in critical illness, where inflammation is believed to be a major driver.^9,22^ This is a potentially modifiable risk factor and may be especially important in resource-limited settings where the incidence of hospital-acquired infection is particularly high, adding further justification for improved infection control and preventative measures.^29,30^

In addition to those already discussed above, our study has several additional limitations. Use of the SF-36 in outcome measurement is limited by its construction and validation largely in Western populations, thus it may not be sensitive to cultural effects in our setting. While patients >70 y of age experienced worse outcomes than younger individuals, a lack of Vietnamese normative data precludes comparison with the wider population and our cohort may have normal values for their age group.

In risk factor evaluation, limited data were available on pre-existing treatment. While no patients were treated with steroids during their ICU stay or reported routine use beforehand, steroids can be components of traditional remedies and it is unknown how many may have been taking these prior to admission.

We have not carried out health-economic evaluations in this study. As the majority of our patients were working-age men, the loss of household income may have serious consequences for patients, families and local communities. A lack of rehabilitation facilities and no community follow-up services means that the burden of care is likely to remain with family members following hospital discharge, further compounding the social and economic impacts of the disease. In designing strategies to improve functional recovery after tetanus, future work should also include health-economic assessment and qualitative evaluation to better understand the economic and emotional consequences of ongoing disability following hospital discharge.

In conclusion, we report that patients with tetanus suffer significant muscle wasting that is associated with worse functional outcome. The effect of muscle wasting on the mental health of survivors warrants further investigation. Costly Western-style multidisciplinary models of care are unfeasible in our setting and alternative solutions are required. Innovative approaches to provide sustainable but short-course training to physiotherapists in resource-limited ICUs have already been piloted and experience from these may be of value in designing future interventions in rehabilitation.^31^ In addition, hospital-acquired infection is a potentially modifiable risk factor for muscle wasting in patients and efforts should continue to reduce the incidence of infection.

## Supplementary Material

trz055_Supplementary_Figure_1

trz055_Supplementary_Figure_2

trz055_Supplement_Tables
