# Supplementary figures and images for: Functional outcome and muscle wasting in adults with tetanus
Source: Trans R Soc Trop Med Hyg. 2019 Jul 24;113(11):706–13. doi: 10.1093/trstmh/trz055 (PMC6836715; doi:10.1093/trstmh/trz055)

Supplementary Figure 1 : Study Flowchart

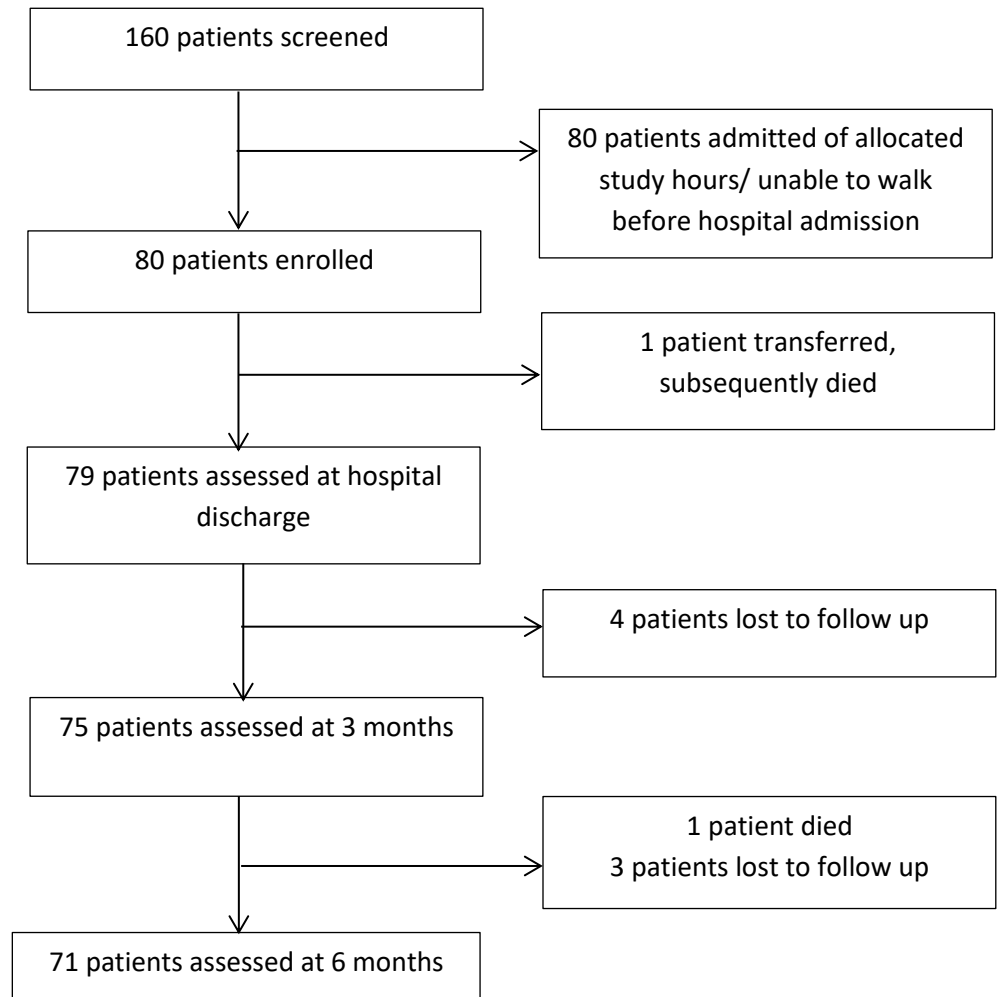

Supplement: trz055_Supplementary_Figure_1 [file trz055_supplementary_figure_1.pdf]
