# Supplementary material for: Functional outcome and muscle wasting in adults with tetanus
Source: Trans R Soc Trop Med Hyg. 2019 Jul 24;113(11):706–13. doi: 10.1093/trstmh/trz055 (PMC6836715; doi:10.1093/trstmh/trz055)

Supplementary Figure 2: RF-CSA (all patients). Linear mixed effects model shown in blue; grey shading showing 95% CI.

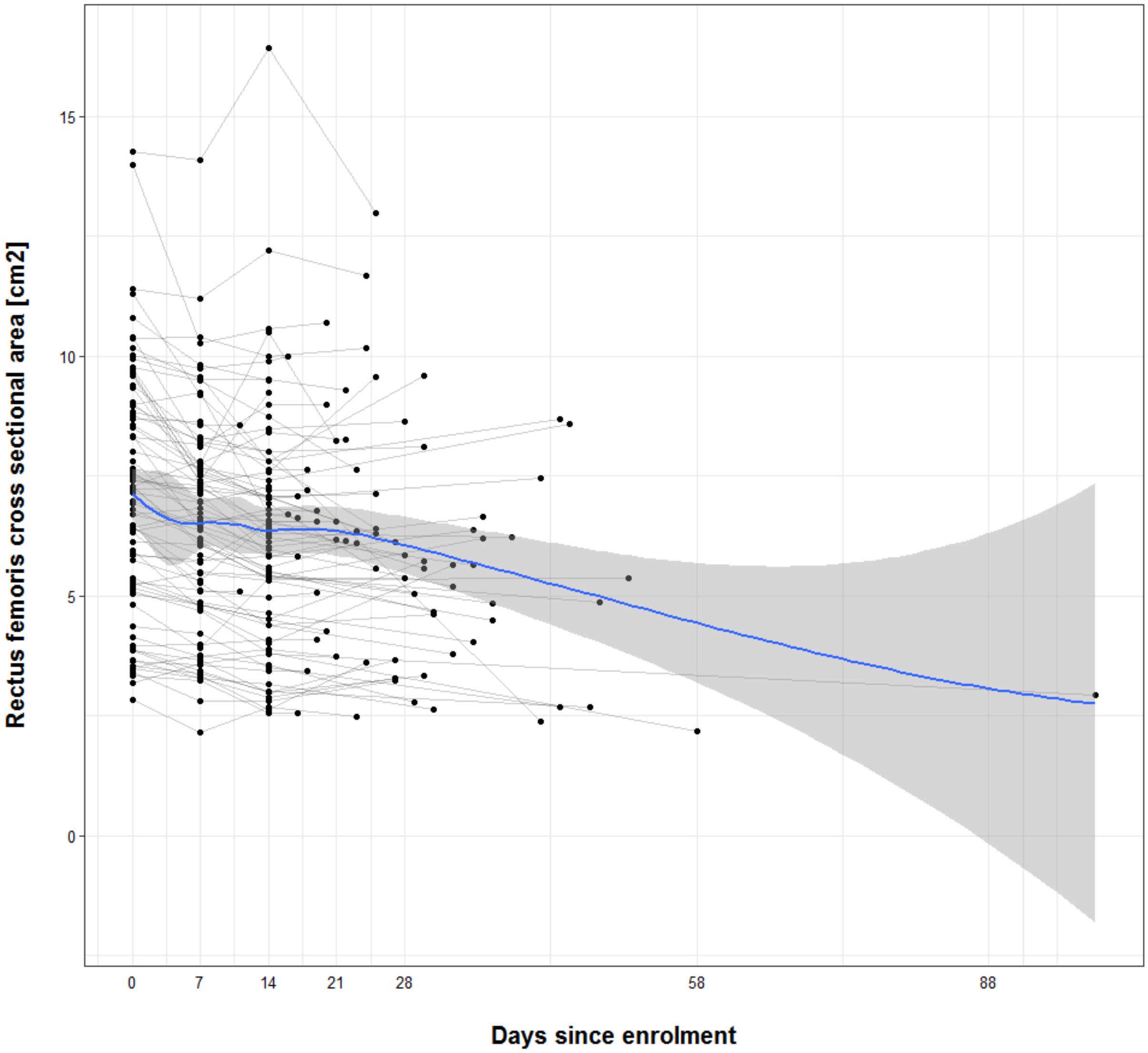

Supplement: trz055_Supplementary_Figure_2 [file trz055_supplementary_figure_2.pdf]
