# Supplementary material for: Functional outcome and muscle wasting in adults with tetanus
Source: Trans R Soc Trop Med Hyg. 2019 Jul 24;113(11):706–13. doi: 10.1093/trstmh/trz055 (PMC6836715; doi:10.1093/trstmh/trz055)
Supplement: trz055_Supplement_Tables [file trz055_supplement_tables.docx]

Supplementary Table 1: Cronbach’s Alpha for SF-36 at hospital discharge, 3 and 6 months after discharge

| Domain | Discharge | 3 month | 6 months |
| --- | --- | --- | --- |
| Physical Function | 0.93 | 0.88 | 0.91 |
| Physical Role Limitation | 0.97 | 1 | 1 |
| Emotional Wellbeing | 0.76 | 0.82 | 0.94 |
| Emotional Role Limitation | 0.98 | 0.99 | 1 |
| Energy | 0.8 | 0.82 | 0.91 |
| Pain | 0.63 | 0.93 | 0.89 |
| General Health | 0.84 | 0.87 | 0.95 |
| Social Function | 0.62 | 087 | 0.81 |

Cronbach’s alpha indicating internal consistency of reliability of test scores. It is generally accepted that a Chronbach’s alpha > 0.7 indicates good reliability.

Supplementary Table 2 : Association between functional outcome and RF-CSA at hospital discharge.

|  | Coefficient* | 95% CI | P |
| --- | --- | --- | --- |
| Frailty | -0.90 | (-1.15; -0.65) | <0.01 |
| TUG | -0.11 | (-0.17; -0.05) | <0.01 |
| Barthel | 0.05 | (0.03; 0.07) | <0.01 |
| SF-36 |  |  |  |
| Physical functioning | 0.04 | (0.02; 0.06) | <0.01 |
| Role limitation due to physical health | 0.01 | (-0.01; 0.03) | 0.48 |
| Role limitation due to emotional problems | 0.004 | (-0.012; 0.02) | 0.58 |
| Energy/ fatigue | 0.03 | (0.01; 0.05) | 0.05 |
| Emotional well-being | 0.003 | (-0.04; 0.04) | 0.84 |
| Social functioning | 0.04 | (0.020; 0.06) | <0.01 |
| Pain | 0.02 | (-0.02; 0.06) | 0.17 |
| General health | 0.06 | (0.04; 0.08) | <0.01 |
| Health change | 0.03 | (0.01; 0.05) | 0.03 |

*Coefficient = regression coefficient representing change in RF-CSA in cm^2^ following 1 unit increase in the variable score (as detailed in Table 3). For example, a 1 unit increase in Clinical Frailty scale is associated with a 0.9 cm^2^ decrease in RF-CSA, a 1 unit increase in Barthel score is increased in a 0.05 cm^2^ increase in RF-CSA at hospital discharge. 95% CI = 95% confidence interval

Supplementary Table 3: Association between functional outcome 6 months after discharge and RF-CSA at hospital discharge.

|  | Coefficient | 95% CI | P |
| --- | --- | --- | --- |
| Barthel | 0.11 | (0.06; 0.17) | 0.05 |
| SF-36 |  |  |  |
| Physical functioning | 0.05 | (0.03; 0.07) | <0.01 |
| Role limitation due to physical health | 0.02 | (0.009; 0.04) | <0.01 |
| Role limitation due to emotional problems | 0.02 | (0.009; 0.04) | <0.01 |
| Energy/ fatigue | 0.06 | (0.04; 0.08) | <0.01 |
| Emotional well-being | 0.07 | (0.031; 0.11) | <0.01 |
| Social functioning | 0.03 | (0.01; 0.05) | 0.09 |
| Pain | 0.07 | (0.03; 0.11) | <0.01 |
| General health | 0.06 | (0.04; 0.08) | <0.01 |
| Health change | 0.06 | (0.04; 0.08) | <0.01 |

*Coefficient = regression coefficient, representing change in RF-CSA in cm^2^ following 1 unit increase in the variable (as detailed in Table 3). For example, a 1 unit increase in Barthel score is increased in a 0.11 cm^2^ increase in RF-CSA at hospital discharge. 95% CI = 95% confidence interval
